# Among Patients with Sustained Viral Suppression in a Resource-Limited Setting, CD4 Gains Are Continuous Although Gender-Based Differences Occur

**DOI:** 10.1371/journal.pone.0073190

**Published:** 2013-08-27

**Authors:** Joseph B. Sempa, Agnes N. Kiragga, Barbara Castelnuovo, Moses R. Kamya, Yukari C. Manabe

**Affiliations:** 1 Infectious Diseases Institute, Makerere University, Kampala, Uganda; 2 Makerere University College of Health Sciences, Kampala, Uganda; 3 Johns Hopkins University, School of Medicine, Baltimore, Maryland, United States of America; University of Alabama at Birmingham, United States of America

## Abstract

**Introduction:**

There is conflicting data on long-term CD4 immune recovery after combination antiretroviral therapy (ART) in resource-limited settings. Virologic suppression is rarely documented in cohorts from sub-Saharan Africa so objective evidence of adherence is biologically unsubstantiated. We sought to investigate long-term patterns of immune recovery in Ugandan patients on ART with sustained viral suppression.

**Methods:**

A prospective cohort of patients starting ART between April, 2004 and April, 2005 at the Infectious Diseases Institute with sustained viral suppression (viral load ≤400 copies/ml at month 6 and 12) while on first-line ART. Propensity scores were used to adjust for treatment allocation (nevirapine or efavirenz) at ART initiation. Data were analyzed using Kaplan Meier methods and cross-sectional time series regression.

**Results:**

Three hundred and fifty-six patients were included in the analysis.71.6% were female, 87% in WHO stage 3 or 4, median age was 37 years, (IQR:32–43), and median CD4 count was 108 cells/µL, (IQR:35–174) at ART start. At multivariable analysis, lower immune recovery (measured by change in CD4 from ART start at each time interval) was associated with male-gender (-59, 95% CI: 90, -28, *P*<0.001), baseline CD4 count of 101–200 cells/µL (-35, 95% CI: 62, -9, *P*=0.009) and >200 (-64, 95% CI: 101, -26, *P*=0.001), and use of AZT at baseline (-47, 95% CI: -74, -20, *P*=0.001). Median time to reach >400 cells/µL was longer in males (197.4 weeks, IQR:119.9–312.0), compared to females (144.7 weeks, IQR:96.6–219.7, *P*<0.001). The cumulative probability of attaining CD4 >400 cells/µL over 7 years was higher in females compared to males (*P*<0.001).

**Conclusions:**

There was long-term, continuous, immunologic recovery up to 7 years after ART initiation in an urban Ugandan cohort. Virologically suppressed women had better sustained immune recovery than men. Men take longer to immune reconstitute and have a lower probability of reaching a CD4 cell count >400 cells/µL. The biologic mechanisms of these gender differences need further exploration.

## Introduction

Potent combination antiretroviral therapy (ART) leads to suppression of the viral load, and immune reconstitution, usually defined by an increase in CD4+ T cell (CD4) count, and consequently, to a reduction in occurrence of AIDS and mortality [1]. However, increases in CD4 counts vary by region [2,3], gender with a higher increase in CD4 count in women [3,4], the duration of antiretroviral therapy [3,5,6] and on the co-existence of other infections, particularly tuberculosis [7]. The greatest increases in CD4 count occur during the first year of ART initiation [4,5,8,9,10], especially in patients with low baseline CD4 count [11]. However patients with low CD4 count are more likely to take longer to return to normal CD4 count levels [5], and may remain at increased risk of opportunistic infection hence morbidity [12]. Two studies in resource-rich countries with a minimum follow-up time of 6 years have shown a significant increase in CD4 count among patients who initiated ART at lower CD4 count level compared to those who started ART at high CD4 count [4,9]. However some patients tend to have a suboptimal and lower rate of immune recovery even if they achieve viral suppression [13] and they remain at increased risk for opportunistic infections and AIDS-related mortality [2].

A few studies have been done in the area of immune recovery in the Sub-Saharan Africa (SSA) but there are no specific data for patients with sustained viral suppression [3,6] since viral load testing is not routinely performed due to the high cost [14]. We sought to investigate long-term patterns of immune reconstitution after ART for up to 7 years, and factors associated with higher CD4 count increase in patients with documented sustained viral suppression.

## Methods

### Study setting and population

The Infectious Diseases Institute (IDI) of Makerere University is a center of excellence which delivers HIV treatment and care to over 10,000active HIV patients with over 8,000 patients on antiretroviral therapy. Nested within the clinic at IDI is a prospective cohort study of 559 patients who started ART between April 2004 and April 2005 and have been followed for over 8 years. Patients were started on stavudine, lamivudine and nevirapine (provided by Global Fund) or zidovudine plus lamivudine plus efavirenz (provided by the US President’s Emergency Plan for AIDS Relief) according to the WHO [15], and national guidelines [16]. Details of this cohort have been previously described in detail [17,18]. Briefly, the study participants have clinic visits every 3 months. Laboratory tests are performed every 6 months, which include CD4 count by FACSCalibur (Becton Dickson, Mountain View, California, USA) and HIV-1 viral loads (Amplicor HIV-1 Monitor PCR Test, version 1.5, Roche Diagnostics, GmbH Molecular Systems, Pleasanton, California, USA, with a lower limit of detection of 400 copies/ml).

This analysis included all patients who had viral load measurements <400 copies/ml at month 6 and 12 and who were taking first-line ART for up to 7years of follow-up; patients were included in the analysis for only the time that they had sustained viral suppression defined as viral load ≤400 copies/μL while still on first-line treatment. Patients were categorized in 3 groups depending on the CD4 count at ART start: 1) ≤100 cells/µL, 2) 101-200 cells/µL, and 3) >200 cells/µL.

### Ethics statement

This study was approved by the institutional review board of Makerere University College of Health Sciences and the Uganda National Council for Science and Technology (MV853). All patients were counseled and provided a written informed consent.

### Statistical analysis

We calculated the increase in CD4 count from baseline to all subsequent 6-monthly CD4 count readings. To obtain unadjusted estimates for the association between change in CD4 count from baseline and each predictor, we used cross-sectional time series regression while controlling for time interval when the laboratory tests were done. Factors considered in the analysis were gender, age, baseline viral load (<5 log_10_copies/ml and ≥5 log_10_copies/ml), and WHO staging (stage 1/2 and stage 3/4). Fortime-updated covariates, we considered time intervals of the CD4 count measurement, weighted estimates of baseline ART regimen, and hemoglobin in the analysis. Predictors with a *P*-value ≤0.2 in the unadjusted analysis and those of clinical significance were included in the multivariable analysis. A multivariable time series regression model was used to determine factors associated with CD4 count increases in these patients. We calculated the time to reach and the probability of achieving a CD4 count >400 cells/µL, which is the lower normal limit of CD4 count in Ugandan adults [19] using the Kaplan Meier method. Curves were compared using the log rank test. We also compared the proportions of mortality and opportunistic infections that occurred during the study period in the 3groups using the chi-square test. Since baseline ART treatment was not randomly assigned, we accounted for the selection bias using the predicted probability of treatment allocation (stavudine plus lamivudine plus nevirapine, and zidovudine plus lamivudine plus efavirenz), and generated propensity scores.

Another sub-analysis was done to study the differences in immunological recovery between male and female gender. We compared the mean age between males and females using a Student’s T-test. We used a chi-square test for trend to analyze for differences in baseline BMI (underweight, normal, and overweight), and baseline CD4 count groups categorized by gender. Chi-square test was used to determine the association between gender and other baseline variables (i.e. regimen, WHO stage, hemoglobin, and HIV RNA viral load). We calculated the time to reach and the probability of achieving a CD4 count >400 cells/μL stratified by gender using the Kaplan Meier method. Wilcoxon rank sum test was used to compare median time, and curves were compared using the log rank test. A *P*<0.05 was considered statistically significant.

Data management and statistical analyses were performed using Stata (StataCorp. STATA 11.1, College Station, Texas 77845 USA)

## Results

**Figure 1:**
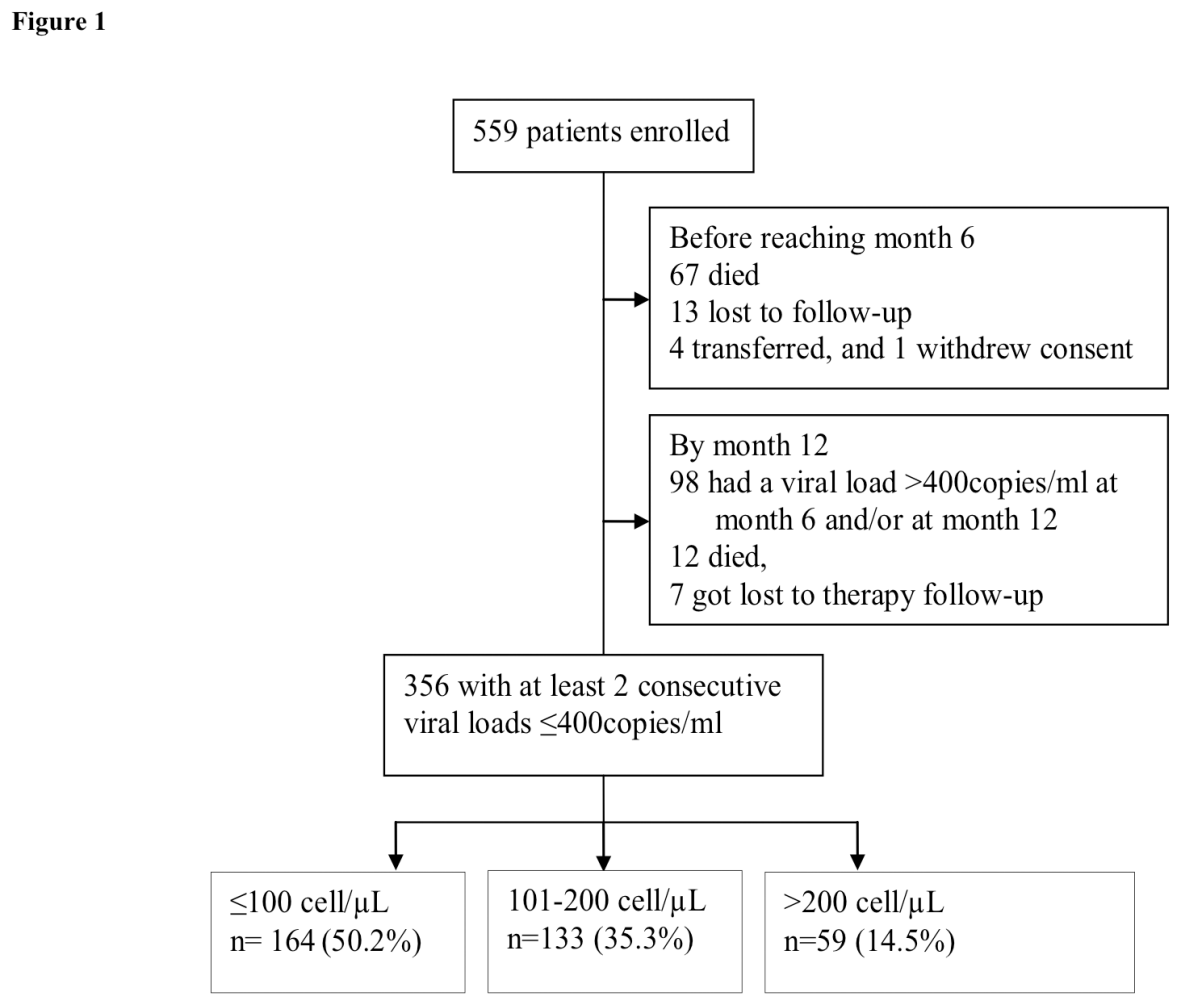
Study flow diagram.

Three hundred fifty-six patients out of the 559 enrolled, were eligible for our study. We excluded202 patients by month 12 (98 had viral load >400copies/ml month 6 and/or 12,79 died, 20 got lost to follow up, 4 were transferred out, and one withdrew consent before reaching 12 months), and one was excluded because of lack of a viral load result at month 6 [20]. The treatment outcomes of our cohort at 6 months of follow up have been described elsewhere [18] (Figure 1).

Of the remaining 356 patients 71.6% were female, 87% had WHO stage 3 or 4, the median age at ART start was 37 years, (IQR: 32-43), and the medianCD4 count at ART start was 108 cells/µL, (IQR 35-174); 72% were started on stavudine plus lamivudine plus nevirapine, and 28% on zidovudine plus lamivudine plus efavirenz. The baseline characteristics of the patients included in the analysis were similar to those of the patients excluded (data not shown). Of the patients included 164 (46%) started ART with a CD4 count ≤100 cells/µL, 133 (37%) with a CD4 count 101-200 cells/µL, and 59 (17%) with a CD4 count >200 cells/µL. Gender distribution, hemoglobin, and ART regimen at start were similar across the three groups except for viral load which was <5 log_10_copies/ml in a higher proportion of patients with a CD4 count >200 cells/µL at ART start, and median age which was higher in the same group. Although the difference was only borderline significant, there were more patients in WHO stage 3 and 4 in the group of patients who started ART with a CD4 count ≤100 cells/µL (Table 1).

**Table 1:** Characteristics of the study participants categorized by CD4 counts at ART initiation (N=356).

**Variable**	**<=100**	**101-200**	**>200**	**P-value**
**Gender**: Male, n(%)	52 (31.7)	35 (26.3)	14 (23.7)	0.406
**Age in years: median(IQR)**	34 (29-40)	36 (32-41)	37 (31-45)	**0.047**
**ART regimen**:n(%)				
Stavudine/lamivudine/nevirapine	118 (72.0)	93 (69.9)	46 (78.0)	
Zidovudine/lamivudine/efavirenz	46 (28.0)	40 (30.1)	13 (22.0)	0.516
**WHO stage**:n(%)				
Stage I-II	13 (7.9)	21 (15.8)	10 (17.0)	
Stage III-IV	151 (92.1)	112 (84.2)	49 (83.1)	0.062
**Hemoglobin**:n(%)				
≤8g/dL	7 (4.3)	4 (3.0)	2 (3.4)	
>8 g/dL	157 (95.7)	129 (97.0)	57 (96.6)	0.841
**HIV RNA Viral load**:n(%)				
<5 log_10_copies/ml	27 (16.5)	32 (24.1)	27 (45.8)	
≥5 log_10_copies/ml	137 (83.5)	101 (75.9)	32 (54.2)	**<0.001**

Note: ART: Antiretroviral therapy; WHO: World Health Organization; n = number, IQR = interquartile range

### Clinical outcomes

Twenty-four patients died during follow-up with a mortality rate that was similar for both males 5 (5%) and females 19 (7.5%) (*P*=0.396). Mortality was higher in patients who had a baseline CD4 count ≤100 cells/µL, 13 (7.9%) compared to patients who had a baseline CD4 count 101-200 cells/µL 7 (5.3%) (*P*=0.0001), and >200 cells/µL 4 (6.8%) (*P*=0.0001). Sixteen patients were lost to follow-up (n=7) or transferred (n=9) with no observed differences by gender. Only one woman withdrew her consent. The overall median time of follow up was 312 weeks (192–336), and was similar across the CD4 categories. One hundred and thirty-six patients(38.2%) were censored during follow up because they had a viral load measurement >400 copies/ml. The mean (SD) follow up time among those who were censored was 185 weeks(7 weeks) and this was similar across gender(*P*=0.285).

### Immunological outcomes

**Figure 2:**
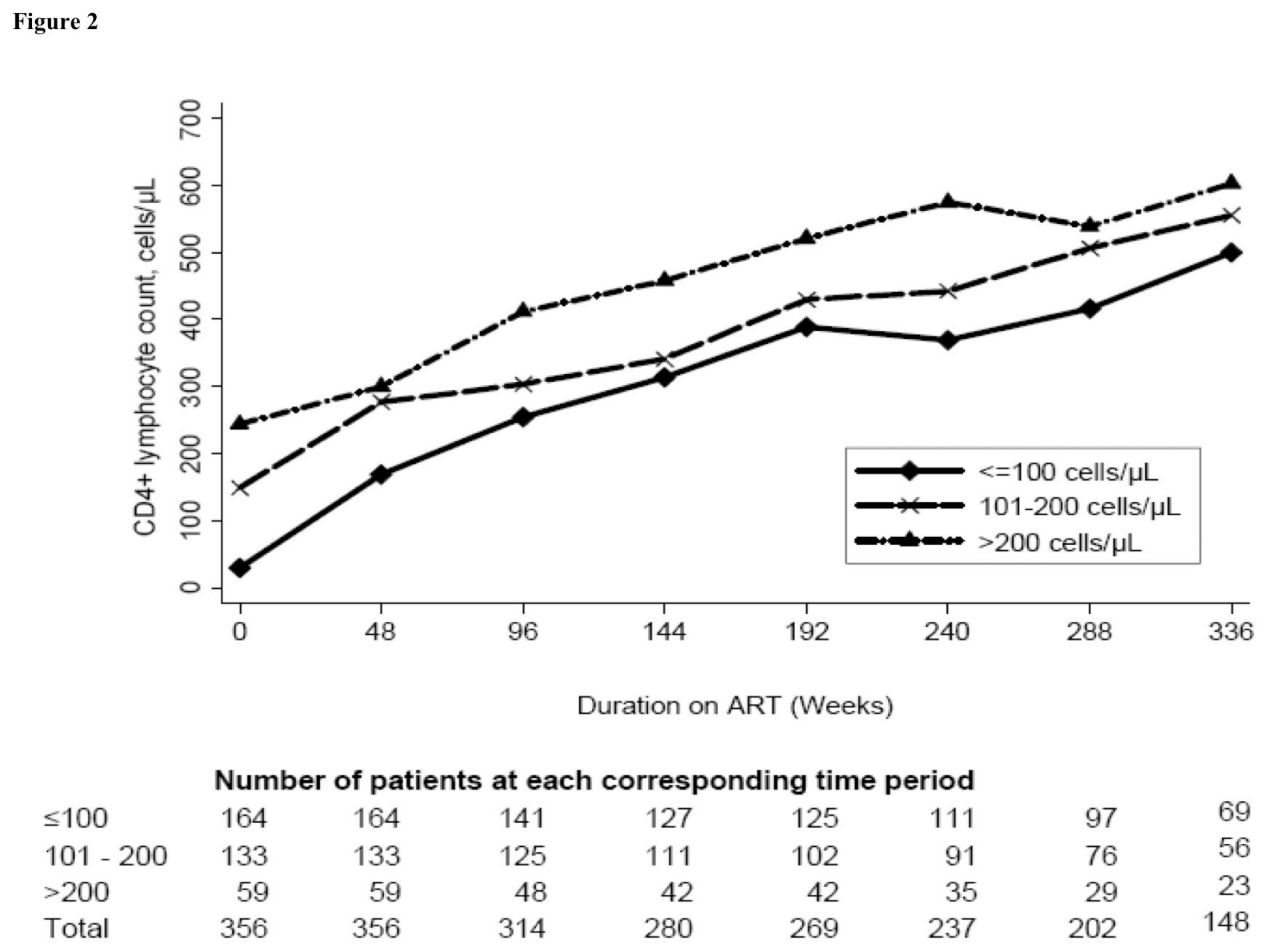
Median CD4 count over time stratified by baseline CD4 count. The full line with diamonds represents people with baseline CD4 count ≤100 cells/µL. The long dash line with ‘x’ s represents people with baseline CD4 count 101-200 cells/µL. The long dash-dotted line with triangles represents people with baseline CD4 count >200 cells/µL.

Overall, the median CD4 count increased from 108 cells/µL (IQR: 35-174) at baseline to 534 cells/µL (IQR: 389, 717) at 7 years (*P*<0.001). The median increase (IQR) during follow-up among those who had ≤100 cells/µL at ART start was 478 cells/µL (IQR: 310, 606) and was significantly higher compared to those with baseline CD4 count of 101-200 cells/µL, (390 cells/µL (IQR:304, 591) (*P*=0.044)), and to those with >200 cells/µL (343 cells/µL (IQR: 118, 496) (*P*=0.008). During the first year of ART, the increase in CD4 count was higher in patients with baseline CD4 count ≤100 cells/µL (136 cells/µL, IQR: 80, 211) compared to those with baseline CD4 count of 101-200 cells/µL, (119 cells/µL, IQR:37, 205, *P*=0.036), and to those who started ART with >200 cells/µL (47 cells/µL, IQR: 0, 122, *P*<0.001). Annual median CD4 count trajectories are shown in detail in Figure 2. Overall, the trajectories of the median CD4 count continued to rise significantly in all the groups up to 7years after ART initiation (Figure 2).

**Figure 3:**
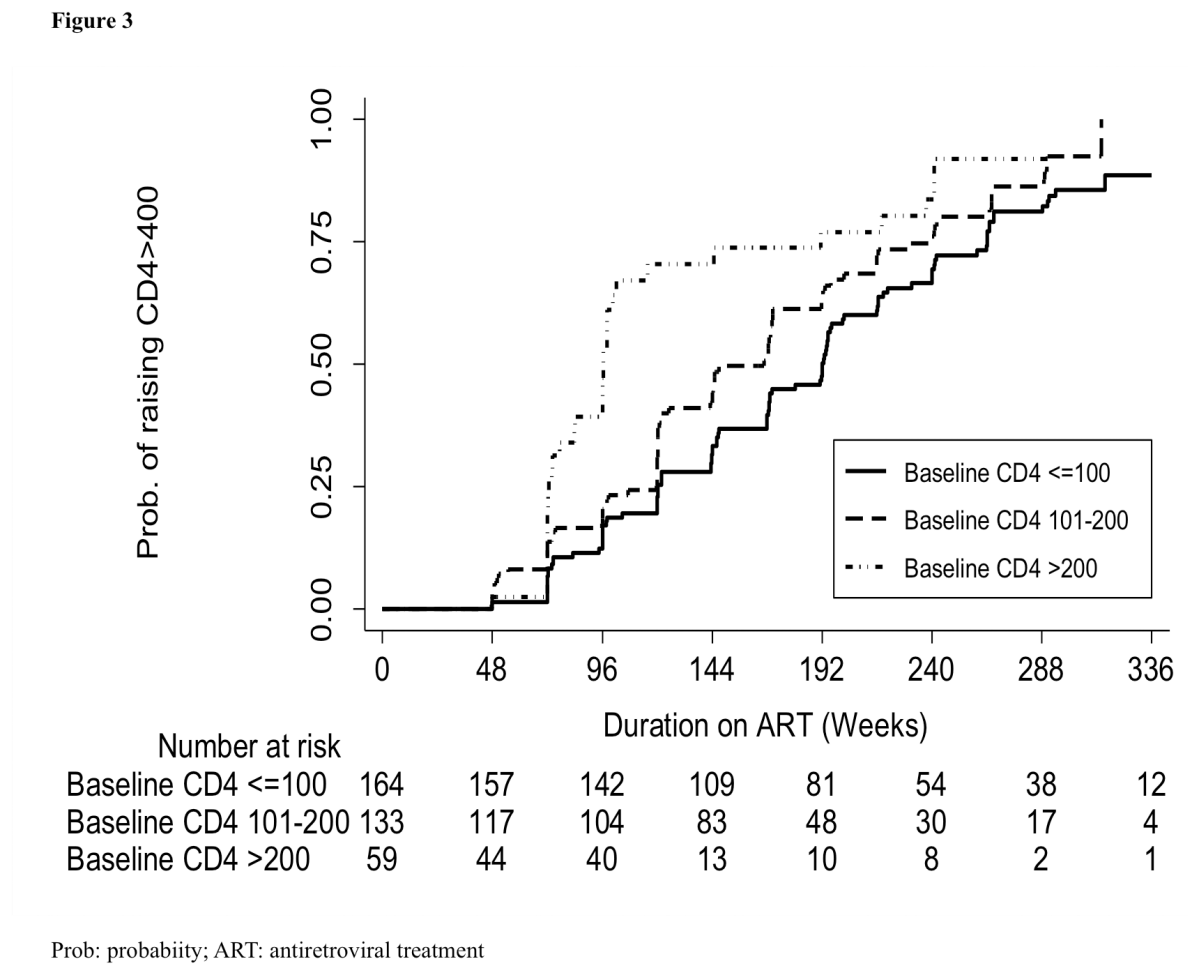
Probability of achieving CD4 count >400 cells/µL and number at risk by baseline CD4 count. The full line represents people with baseline CD4 count ≤100 cells/µL. The long dash line represents people with baseline CD4 count 101-200 cells/µL. The long dash-dotted line represents people with baseline CD4 count >200 cells/µL.

As expected the probability of attaining a CD4 >400 cells/µL across the 3 groups was lower in patients who started ART at CD4 count ≤100 cells/µL (87.4%,95% CI78.4,93.9) compared to those started at 101-200 cells/µL (94.4%, 95% CI85.3,98.7, *P*=0.018), and >200 cells/µL (88.9, 95% CI75.0,97.0, P<0.001) (Figure 3). The median time to reach >400 cells/µL was longer in patients with baseline CD4 count of ≤100 cells/µL (1,183 days, IQR 839, 1,531), compared with patients with CD4 count of 101-200 cells/µL (939 days, IQR 673, 1354), and >200 cells/µL (672 days, IQR: 506, 710, *P*<0.001).

### Risk factors for lower immune recovery

The bivariate analysis (Table 2) showed that, age, male gender, and baseline CD4 count >200 and 101-200 cells/µL compared to ≤100 cells/µL were associated with an annual CD4 count reduction of-12, -48, -41, and -82cells/μL respectively. Patients with a baseline viral load of ≥5 log_10_copies/ml had higher rates of immune recovery compared to patients with < 5 log_10_copies/ml at ART start.

**Figure 4:**
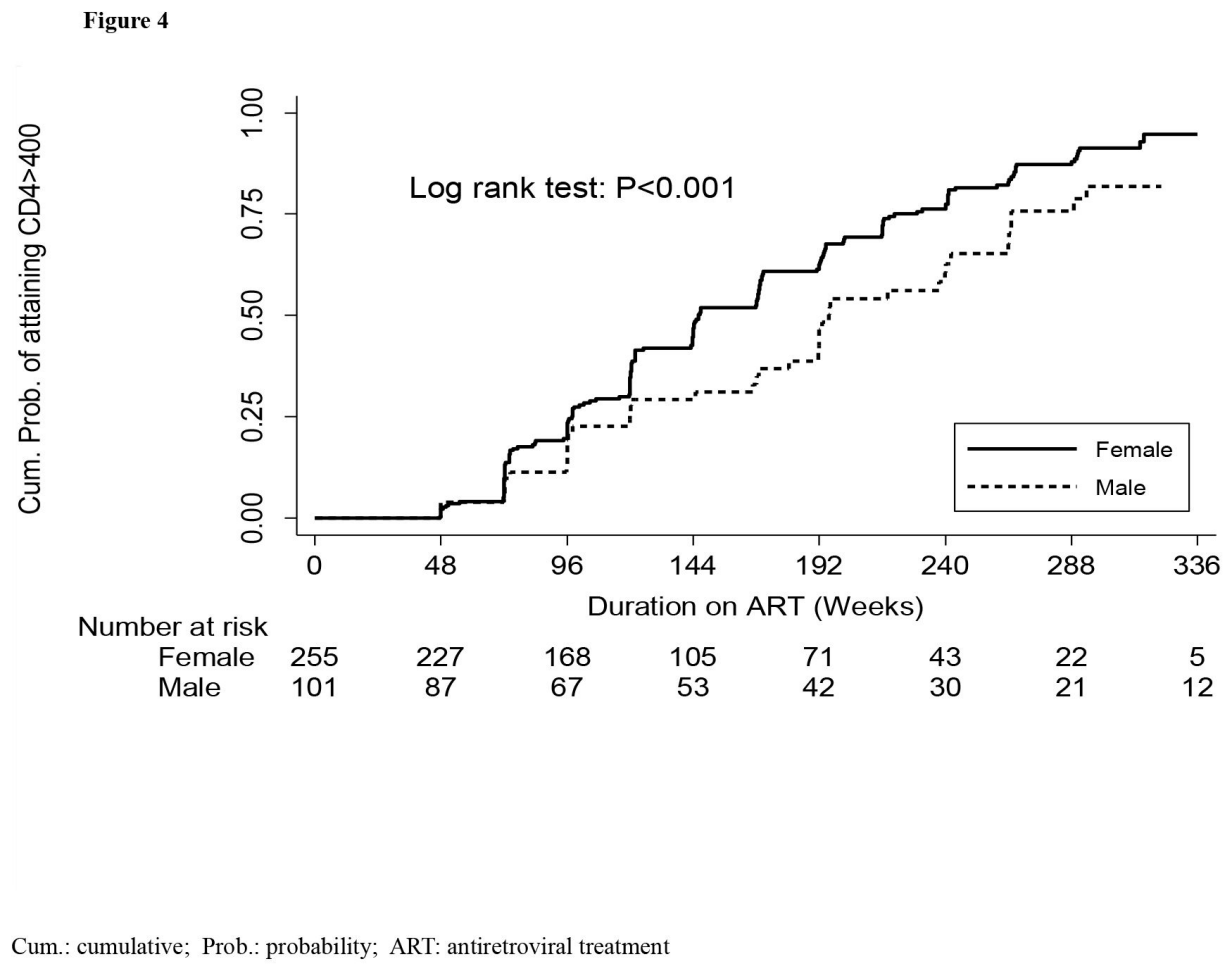
Probability of achieving CD4 count normalization (i.e. CD4 >400 cells/µL) and number at risk by gender. The full line represents women. The long dash line represents men.

**Figure 5:**
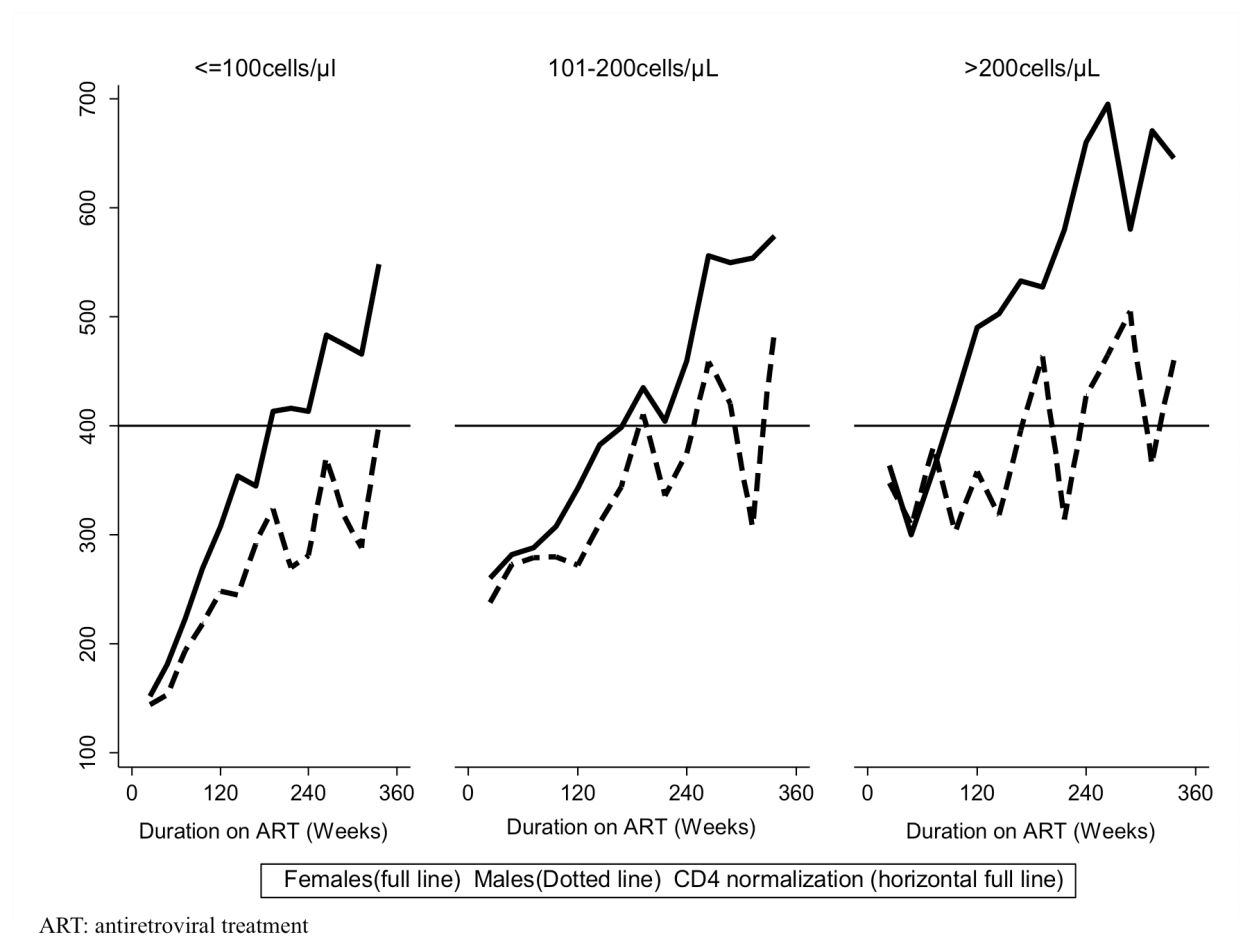
Median CD4 count trajectory stratified by gender within baseline CD4 count groups. The full line represents women. The long dash line represents men.

**Table 2:** Cross-sectional time series regression model of factors associated with lower immune recovery (N=356).

**Variable**	**Unadjusted model**				**Adjusted model**		
	Change in CD4 count	95% C.I.	P-value		Change in CD4 count	95% C.I.	P-value
*Baseline* *:*							
Age (5-year increase)	-12	-18, -4	0.003		-5	-12,3	0.206
Gender, Male	-48	-77, -19	0.001		-59	-90, -28	**<0.001**
CD4 count: cells/μL							
≤ 100	Reference				Reference		
101-200	-41	-69, -13	0.004		-36	-63, -9	**0.009**
>200	-82	-119, -45	<0.001		-64	-102, -26	**0.001**
HIV RNA Viral load							
<5 log_10_copies/ml	Reference				Reference		
≥5 log_10_copies/ml	52	21,83	0.001		46	17, 76	**0.002**
Allocation of NNTRI based regimen^**^	65	-179, 309	0.600		162	-103, 427	0.232
AZT based ART regimen					-47	-74, -20	**0.001**
*Time* *updated* *:*							
Follow-up time (years)	52	50, 55	<0.001		53	50, 55	**<0.001**
Hemoglobin: >8g/dl	44	-10,98	0.113		41	-13, 95	0.134

** NNTRI based regimen (efavirenz or nevirapine) was analyzed using propensity scores

Note: WHO stage, hemoglobin at ART initiation, Body Mass Index (BMI) and opportunistic infections were not associated with immune recovery at bivariate and multivariable analysis.

CI = confidence interval, n = number, ART = antiretroviral therapy, NNRTI = non-nucleoside reverse transcriptase inhibitor, AZT = zidovudine

In the multivariable analysis, factors associated with lower yearly immune recovery were: CD4 count of 101-200 cells/µL at ART start (-36 cells/µL,95%CI: 63, -9, *P*=0.009) and CD4 count >200 cells/µL (-64cells//μL,95%CI:-102, -26, *P*=0.001) compared to those with ≤100 cells/µL at ART start), and male gender (-59 cells/µL, 95%CI: 90, -28, *P*<0.001). Baseline viral load ≥5 log_10_copies/ml was also associated with a higher immune recovery (46 cells/µL,95%CI:17,76, *P*=0.002), per year.

### Immunologic recovery by gender

At baseline, males and females had similar characteristics except that the males were older than women (38 years, IQR:35, 42 versus 34 IQR:29, 40; *P*<0.001) (Table 3). Other characteristics were similar including the median CD4 count at ART initiation within the CD4 count strata: median time to reach >400 cells/µL was longer in males (197weeks, IQR:120-312) compared to females (145, IQR:97, 220) (*P*<0.001). The cumulative probability of attaining CD4 >400 cells/µL during 7year follow-up was also higher among females (92.1%, 95% CI: 90.7, 93.3) compared to males (63.9% (95%CI: 60.5-67.3) (*P*<0.001) (Figure 4). The women had also a higher immune recovery compared to men across all the CD4 count strata (Figure 5).

## Discussion

The CD4 count of patients with sustained viral suppression enrolled in a prospective cohort in Uganda continues to rise over 7years of follow-up, similar to other analyses from cohorts in Europe and North America with a follow up of ≥ 6 years [11,21]. In a UK cohort with a relatively high proportion(2056/7069, 29.1%) of Black Africans, CD4 counts continued to rise in the 8years of follow-up among patients with viral load consistently <1000copies/ml without attaining a plateau regardless of CD4 count at initiation [22]. Our study findings were also similar to other sub-Saharan African cohorts in Malawi, Uganda, and Kenya where patients had continued CD4 gains up to 6 years on ART, although the authors did not limit their analysis to patients with sustained viral suppression and patients had generally higher baseline CD4 counts compared to patients in our cohort [23]. In contrast, some studies have shown an initial increase in CD4 count and a leveling off after 4years of follow-up [9,24]. The level of CD4 counts in patients on ART reached a plateau, especially in those with higher CD4 cell counts at baseline [4,25,26].

**Table 3:** Characteristics of study participants stratified by gender at ART initiation.

**Variable**	**Female n=255**	**Male n=101**	**Total n=356**	**P-value**
**Age in years**; median(IQR)	34 (29-40)	38 (35-42)	35 (30-42)	<0.001^*^
Body mass Index; Kg/m^2^				0.113^***^
<18.5	69 (27.0)	35 (34.7)	104 (29.2)	
18.5-24.9	160 (62.8)	59 (58.4)	219 (61.5)	
≥25	26 (10.2)	7 (6.9)	33 (9.3)	
**CD4 cell count** (cells/μL)**;**n(%)				0.181^***^
≤100	112 (43.9)	52 (51.4)	164 (46.0)	
101-200	98 (38.4)	35 (34.7)	133 (37.4)	
>200	45 (17.7)	14 (13.9)	59 (16.6)	
**Regimen**:n(%)				0.305^**^
Nevirapine-based	188 (73.7)	69 (68.3)	257 (72.2)	
Efavirenz-based	67 (26.3)	32 (31.7)	99 (27.8)	
**WHO stage**:n(%)				0.05^**^
1/2	37 (14.5)	7 (6.9)	44 (12.4)	
3/4	218 (85.5)	94 (93.1)	312 (87.6)	
**Hemoglobin** (g/dl)**;**n(%)				0.411^**^
≤8	8 (3.1)	5 (5.0)	13 (3.7)	
>8	247 (96.9)	96 (95.0)	343 (96.3)	
**HIV RNA** log_10_copies/ml**;**n(%)				0.227^**^
<5	66 (25.9)	20 (19.8)	86 (24.2)	
≥5	189 (74.1)	81 (80.2)	270 (75.8)	

^*^ p-value from t-test; ^**^ p-value for chi-square test; ^***^p-value for chi-square test for trend

IQR = interquartile range, n = number

In our cohort, the magnitude of the increase in CD4 count after every measurement generally becomes smaller (i.e. CD4 count increases at a decreasing rate) and is most consistent with an asymptote rather than a plateau. This has also been described in other studies [21,27] were patients with higher change in CD4 counts (>200 cells/µL) from ART to 2 years’ experience lower increases after 2years on ART. The increased immune recovery seen in the early stages of ART initiation is mainly due to the decreasing viral load levels of the patients [28,29]. Some authors have suggested that the initial trajectory of CD4 count after ART initiation is due to redistribution of memory CD4 cells [30] from lymphoid tissues towards the blood compartment. This trajectory evolves into an asymptote after long-term ART because of an impaired activity in the bone marrow [31] and/or reduced thymic activity.

Consistent with other studies, patients who started ART at higher CD4 counts >200 cells/µL were more likely to achieve a CD4 count >400 cells/µL [4,9,10,23,25]. This suggests that despite the higher immune recovery seen immediately after initiation of ART among patients with lower CD4 count thresholds, thereis an increased risk of the hazards associated with patients with lower baseline CD4 counts since they remain at a CD4 count <400 cells/μL for a longer time period [32,33,34]. These results have been reported by other studies [12,35]. A possible explanation could be that while CD4 counts remain low, the bone marrow generates IL-7 which is responsible for the increased stimulation of the production of T-cells as well as their proliferation and survival [12], which is increased in lymphopenic conditions [36].

Contrary to other studies [4,9,23,37,38,39], in our analysis older age at initiation was not associated with lower immune recovery which has been attributed to decreasing thymic reserves [40,41]. Notwithstanding the differences in the definition of viral suppression, our findings were consistent with another study in Senegal with median age 37 years that did not demonstrate an effect of age on immune reconstitution [27].

Male gender was associated with lower immune recovery compared to the female gender. Previous studies have also attributed the difference to higher physiological CD4 count levels in females compared to males [23,42,43] and lower CD4 counts at ART start in males [44]. In a multi-center analysis of patients on ART from 27 sites largely in resource limited settings, slower immune recovery was found in men compared to women [6]. It has been suggested that gender differences could be as a result of other unmeasured confounders including hormones. Studies in resource rich settings have shown that female hormones may stimulate increased production of CD4 T cells compared to male hormones [45,46]. Another explanation may be that the thymic output is higher in women than in men; however, studies to definitely show this are lacking [35].

In conclusion, patients who start ART at lower CD4 count have the greatest CD4 increase in the first years compared to those with higher CD4 counts, however, these patients take longer and are less likely to reach CD4 count normalization. Females have a better immune response and are more likely to achieve earlier CD4 count normalization compared to the males. This study has shown that patients with good adherence to ART as demonstrated by viral suppression have the potential to immune reconstitute beyond normalization, thereby reducing the chances of opportunistic infections and death.

## References

[1] Palella FJ Jr, Delaney KM, Moorman AC, Loveless MO, Fuhrer J et al. (1998) Declining morbidity and mortality among patients with advanced human immunodeficiency virus infection. HIV Outpatient Study Investigators. N Engl J Med 338: 853-860. doi:10.1056/NEJM199803263381301. PubMed: 9516219. 9516219 10.1056/NEJM199803263381301

[2] Geng Elvin, Vittinghoff E, Nachega J, Moore R, Wood R et al (2010) A Comparison of the Immunologic Efficacy of Antiretroviral Therapy in Resource-replete vs Resource-limited Settings. 17th Conference on Retroviruses and Opportunistic Infections. San Francisco

[3] Kesselring AM, Gras L, Wit FW, Smit C, Geerlings SE et al. (2010) Immune restoration and onset of new AIDS-defining events with combination antiretroviral therapy in HIV type-1-infected immigrants in the Netherlands. Antivir Ther 15: 871-879. doi:10.3851/IMP1638. PubMed: 20834099. 20834099 10.3851/IMP1638

[4] Gras L, Kesselring AM, Griffin JT, van Sighem AI, Fraser C et al. (2007) CD4 cell counts of 800 cells/mm^3^ or greater after 7 years of highly active antiretroviral therapy are feasible in most patients starting with 350 cells/mm^3^ or greater. J Acquir Immune Defic Syndr 45: 183-192. doi:10.1097/QAI.0b013e31804d685b. PubMed: 17414934. 17414934 10.1097/QAI.0b013e31804d685b

[5] Tarwater PM, Margolick JB, Jin J, Phair JP, Detels R et al. (2001) Increase and plateau of CD4 T-cell counts in the 3(1/2) years after initiation of potent antiretroviral therapy. J Acquir Immune Defic Syndr 27: 168-175. doi:10.1097/00042560-200106010-00012. PubMed: 11404539. 11404539 10.1097/00126334-200106010-00012

[6] Nash D, Katyal M, Brinkhof MW, Keiser O, May M et al. (2008) Long-term immunologic response to antiretroviral therapy in low-income countries: a collaborative analysis of prospective studies. AIDS 22: 2291-2302. doi:10.1097/QAD.0b013e3283121ca9. PubMed: 18981768. 18981768 10.1097/QAD.0b013e3283121ca9 PMC2794130

[7] Hermans SM, Kiragga AN, Schaefer P, Kambugu A, Hoepelman AI et al. (2010) Incident tuberculosis during antiretroviral therapy contributes to suboptimal immune reconstitution in a large urban HIV clinic in sub-Saharan Africa. PLOS ONE 5: e10527. doi:10.1371/journal.pone.0010527. PubMed: 20479873. 20479873 10.1371/journal.pone.0010527 PMC2866328

[8] Kelley CF, Kitchen CM, Hunt PW, Rodriguez B, Hecht FM et al. (2009) Incomplete peripheral CD4+ cell count restoration in HIV-infected patients receiving long-term antiretroviral treatment. Clin Infect Dis 48: 787-794. doi:10.1086/597093. PubMed: 19193107. 19193107 10.1086/597093 PMC2720023

[9] Moore RD, Keruly JC (2007) CD4+ cell count 6 years after commencement of highly active antiretroviral therapy in persons with sustained virologic suppression. Clin Infect Dis 44: 441-446. doi:10.1086/510746. PubMed: 17205456. 17205456 10.1086/510746

[10] Smith CJ, Sabin CA, Youle MS, Kinloch-de Loes S, Lampe FC et al. (2004) Factors influencing increases in CD4 cell counts of HIV-positive persons receiving long-term highly active antiretroviral therapy. J Infect Dis 190: 1860-1868. doi:10.1086/425075. PubMed: 15499544. 15499544 10.1086/425075

[11] Egger S, Petoumenos K, Kamarulzaman A, Hoy J, Sungkanuparph S et al. (2009) Long-term patterns in CD4 response are determined by an interaction between baseline CD4 cell count, viral load, and time: The Asia Pacific HIV Observational Database (APHOD). J Acquir Immune Defic Syndr 50: 513-520. doi:10.1097/QAI.0b013e31819906d3. PubMed: 19408354. 19408354 10.1097/qai.0b013e31819906d3 PMC2752681

[12] Pinzone MR, Di Rosa M, Cacopardo B, Nunnari G (2012) HIV RNA suppression and immune restoration: can we do better? Clin Dev Immunol, 2012: 2012: 515962. PubMed: 22489250 10.1155/2012/515962 PMC3318265 22489250

[13] Nakanjako D, Kiragga A, Ibrahim F, Castelnuovo B, Kamya MR et al. (2008) Sub-optimal CD4 reconstitution despite viral suppression in an urban cohort on antiretroviral therapy (ART) in sub-Saharan Africa: frequency and clinical significance. AIDS Res Ther 5: 23. doi:10.1186/1742-6405-5-23. PubMed: 18957083. 18957083 10.1186/1742-6405-5-23 PMC2605744

[14] Reynolds SJ, Nakigozi G, Newell K, Ndyanabo A, Galiwongo R et al. (2009) Failure of immunologic criteria to appropriately identify antiretroviral treatment failure in Uganda. AIDS 23: 697-700. doi:10.1097/QAD.0b013e3283262a78. PubMed: 19209067. 19209067 10.1097/QAD.0b013e3283262a78 PMC2720562

[15] World Health Organization (2004) Scaling up antiretroviral therapy in resource-limited settings: treatment guidelines for a public health approach. Geneva: World Health Organization.

[16] Katabira Elly T, Kamya Moses R (2003) National Antiretroviral Treatment and Care Guidelines for Adults and Children. In: Programme SAC, editor. 1st ed. Kampala: Ministry of Health pg 22

[17] Kamya MR, Mayanja-Kizza H, Kambugu A, Bakeera-Kitaka S, Semitala F et al. (2007) Predictors of long-term viral failure among ugandan children and adults treated with antiretroviral therapy. J Acquir Immune Defic Syndr 46: 187-193. doi:10.1097/QAI.0b013e31814278c0. PubMed: 17693883. 17693883 10.1097/QAI.0b013e31814278c0

[18] Castelnuovo B, Manabe YC, Kiragga A, Kamya M, Easterbrook P et al. (2009) Cause-specific mortality and the contribution of immune reconstitution inflammatory syndrome in the first 3 years after antiretroviral therapy initiation in an urban African cohort. Clin Infect Dis 49: 965-972. doi:10.1086/605500. PubMed: 19673615. 19673615 10.1086/605500

[19] Ugandan Reference Ranges (2009). Flow Cyto – Adults. Makerere University Johns Hopkins University(MUJHU) laboratory

[20] Castelnuovo B, Sempa J, Agnes KN, Kamya MR, Manabe YC (2011) Evaluation of WHO Criteria for Viral Failure in Patients on Antiretroviral Treatment in Resource-Limited Settings. AIDS Res Treat, 2011: 2011: 736938. PubMed: 21541225 10.1155/2011/736938 PMC3085383 21541225

[21] Mocroft A, Phillips AN, Gatell J, Ledergerber B, Fisher M et al. (2007) Normalisation of CD4 counts in patients with HIV-1 infection and maximum virological suppression who are taking combination antiretroviral therapy: an observational cohort study. Lancet 370: 407-413. doi:10.1016/S0140-6736(07)60948-9. PubMed: 17659333. 17659333 10.1016/S0140-6736(07)60948-9

[22] Hughes RA, Sterne JA, Walsh J, Bansi L, Gilson R et al. (2011) Long-term trends in CD4 cell counts and impact of viral failure in individuals starting antiretroviral therapy: UK Collaborative HIV Cohort (CHIC) study. HIV Med 12: 583-593. doi:10.1111/j.1468-1293.2011.00929.x. PubMed: 21569188. 21569188 10.1111/j.1468-1293.2011.00929.x

[23] Maman D, Pujades-Rodriguez M, Subtil F, Pinoges L, McGuire M et al. (2012) Gender differences in immune reconstitution: a multicentric cohort analysis in sub-saharan Africa. PLOS ONE 7: e31078. doi:10.1371/journal.pone.0031078. PubMed: 22363550. 22363550 10.1371/journal.pone.0031078 PMC3281917

[24] García F, de Lazzari E, Plana M, Castro P, Mestre G et al. (2004) Long-term CD4+ T-cell response to highly active antiretroviral therapy according to baseline CD4+ T-cell count. J Acquir Immune Defic Syndr 36: 702-713. doi:10.1097/00126334-200406010-00007. PubMed: 15167289. 15167289 10.1097/00126334-200406010-00007

[25] Lok JJ, Bosch RJ, Benson CA, Collier AC, Robbins GK et al. (2010) Long-term increase in CD4+ T-cell counts during combination antiretroviral therapy for HIV-1 infection. AIDS 24: 1867-1876. doi:10.1097/QAD.0b013e32833adbcf. PubMed: 20467286. 20467286 10.1097/QAD.0b013e32833adbcf PMC3018341

[26] Le Moing V, Thiébaut R, Chêne G, Sobel A, Massip P et al. (2007) Long-term evolution of CD4 count in patients with a plasma HIV RNA persistently <500 copies/mL during treatment with antiretroviral drugs. HIV Med 8: 156-163. doi:10.1111/j.1468-1293.2007.00446.x. PubMed: 17461859. 17461859 10.1111/j.1468-1293.2007.00446.x

[27] De Beaudrap P, Etard JF, Diouf A, Ndiaye I, Guèye NF et al. (2009) Modeling CD4+ cell count increase over a six-year period in HIV-1-infected patients on highly active antiretroviral therapy in Senegal. Am J Trop Med Hyg 80: 1047-1053. PubMed: 19478274. 19478274

[28] Staszewski S, Miller V, Sabin C, Schlecht C, Gute P et al. (1999) Determinants of sustainable CD4 lymphocyte count increases in response to antiretroviral therapy. AIDS 13: 951-956. doi:10.1097/00002030-199905280-00011. PubMed: 10371176. 10371176 10.1097/00002030-199905280-00011

[29] Renaud M, Katlama C, Mallet A, Calvez V, Carcelain G et al. (1999) Determinants of paradoxical CD4 cell reconstitution after protease inhibitor-containing antiretroviral regimen. AIDS 13: 669-676. doi:10.1097/00002030-199904160-00007. PubMed: 10397561. 10397561 10.1097/00002030-199904160-00007

[30] Autran B, Carcelaint G, Li TS, Gorochov G, Blanc C et al. (1999) Restoration of the immune system with anti-retroviral therapy. Immunol Lett 66: 207-211. doi:10.1016/S0165-2478(98)00159-X. PubMed: 10203056. 10203056 10.1016/s0165-2478(98)00159-x

[31] Isgrò A, Leti W, De Santis W, Marziali M, Esposito A et al. (2008) Altered clonogenic capability and stromal cell function characterize bone marrow of HIV-infected subjects with low CD4+ T cell counts despite viral suppression during HAART. Clin Infect Dis 46: 1902-1910. doi:10.1086/588480. PubMed: 18462177. 18462177 10.1086/588480

[32] Siddique MA, Hartman KE, Dragileva E, Dondero M, Gebretsadik T et al. (2006) Low CD4+ T cell nadir is an independent predictor of lower HIV-specific immune responses in chronically HIV-1-infected subjects receiving highly active antiretroviral therapy. J Infect Dis 194: 661-665. doi:10.1086/505913. PubMed: 16897665. 16897665 10.1086/505913

[33] Egger M, May M, Chêne G, Phillips AN, Ledergerber B et al. (2002) Prognosis of HIV-1-infected patients starting highly active antiretroviral therapy: a collaborative analysis of prospective studies. Lancet 360: 119-129. doi:10.1016/S0140-6736(02)09411-4. PubMed: 12126821. 12126821 10.1016/s0140-6736(02)09411-4

[34] Hogg RS, Yip B, Chan KJ, Wood E, Craib KJ et al. (2001) Rates of disease progression by baseline CD4 cell count and viral load after initiating triple-drug therapy. JAMA 286: 2568-2577. doi:10.1001/jama.286.20.2568. PubMed: 11722271. 11722271 10.1001/jama.286.20.2568

[35] Corbeau P, Reynes J (2011) Immune reconstitution under antiretroviral therapy: the new challenge in HIV-1 infection. Blood 117: 5582-5590. doi:10.1182/blood-2010-12-322453. PubMed: 21403129. 21403129 10.1182/blood-2010-12-322453

[36] Napolitano LA, Grant RM, Deeks SG, Schmidt D, De Rosa SC et al. (2001) Increased production of IL-7 accompanies HIV-1-mediated T-cell depletion: implications for T-cell homeostasis. Nat Med 7: 73-79. doi:10.1038/83381. PubMed: 11135619. 11135619 10.1038/83381

[37] Lawn SD, Myer L, Bekker LG, Wood R (2006) CD4 cell count recovery among HIV-infected patients with very advanced immunodeficiency commencing antiretroviral treatment in sub-Saharan Africa. BMC Infect Dis 6: 59. doi:10.1186/1471-2334-6-59. PubMed: 16551345. 16551345 10.1186/1471-2334-6-59 PMC1435908

[38] Kaufmann GR, Perrin L, Pantaleo G, Opravil M, Furrer H et al. (2003) CD4 T-lymphocyte recovery in individuals with advanced HIV-1 infection receiving potent antiretroviral therapy for 4 years: the Swiss HIV Cohort Study. Arch Intern Med 163: 2187-2195. doi:10.1001/archinte.163.18.2187. PubMed: 14557216. 14557216 10.1001/archinte.163.18.2187

[39] Hunt PW, Deeks SG, Rodriguez B, Valdez H, Shade SB et al. (2003) Continued CD4 cell count increases in HIV-infected adults experiencing 4 years of viral suppression on antiretroviral therapy. AIDS 17: 1907-1915. doi:10.1097/00002030-200309050-00009. PubMed: 12960823. 12960823 10.1097/00002030-200309050-00009

[40] Maartens G, Boulle A (2007) CD4 T-cell responses to combination antiretroviral therapy. Lancet 370: 366-368. doi:10.1016/S0140-6736(07)60949-0. PubMed: 17659332. 17659332 10.1016/S0140-6736(07)60949-0

[41] Teixeira L, Valdez H, McCune JM, Koup RA, Badley AD et al. (2001) Poor CD4 T cell restoration after suppression of HIV-1 replication may reflect lower thymic function. AIDS 15: 1749-1756. doi:10.1097/00002030-200109280-00002. PubMed: 11579235. 11579235 10.1097/00002030-200109280-00002

[42] Giordano TP, Wright JA, Hasan MQ, White AC Jr., Graviss EA et al. (2003) Do sex and race/ethnicity influence CD4 cell response in patients who achieve virologic suppression during antiretroviral therapy? Clin Infect Dis 37: 433-437. doi:10.1086/376638. PubMed: 12884169. 12884169 10.1086/376638

[43] Sterling TR, Pisell-Noland T, Perez JL, Astemborski J, McGriff JR et al. (2005) Sex-based differences in T lymphocyte responses in HIV-1-seropositive individuals. J Infect Dis 191: 881-885. doi:10.1086/427827. PubMed: 15717262. 15717262 10.1086/427827

[44] Gandhi RT, Spritzler J, Chan E, Asmuth DM, Rodriguez B et al. (2006) Effect of baseline- and treatment-related factors on immunologic recovery after initiation of antiretroviral therapy in HIV-1-positive subjects: results from ACTG 384. J Acquir Immune Defic Syndr 42: 426-434. doi:10.1097/01.qai.0000226789.51992.3f. PubMed: 16810109. 16810109 10.1097/01.qai.0000226789.51992.3f

[45] Molloy EJ, O’Neill AJ, Grantham JJ, Sheridan-Pereira M, Fitzpatrick JM et al. (2003) Sex-specific alterations in neutrophil apoptosis: the role of estradiol and progesterone. Blood 102: 2653-2659. doi:10.1182/blood-2003-02-0649. PubMed: 12791649. 12791649 10.1182/blood-2003-02-0649

[46] Olsen NJ, Kovacs WJ (2011) Evidence that androgens modulate human thymic T cell output. J Investig Med 59: 32-35. PubMed: 21218609. 10.2310/jim.0b013e318200dc98 PMC3077079 21218609

